# Primary Health Institutions and Service Quality in China: Implications for Health Policy

**DOI:** 10.3390/ijerph191912063

**Published:** 2022-09-23

**Authors:** Junfang Xu, Yuyin Zhou, Ruyu Liu, Feng Cheng, Wannian Liang

**Affiliations:** 1 Institute of Social Medicine, School of Public Health, Zhejiang University School of Medicine, Hangzhou 310058, China; 2 Vanke School of Public Health, Tsinghua University, Beijing 100084, China; 3 Institute for Healthy China, Tsinghua University, Beijing 100084, China

**Keywords:** primary health institutions, service quality, health policy

## Abstract

Background: To protect and improve the health of populations, the important role of primary health institutions has been strengthened through a series of health policies, especially the implementation of a national hierarchical diagnosis and treatment system. In this light, we aim to evaluate the development of primary health institutions between 2013, before the implementation of the hierarchical diagnosis and treatment system, and 2020 as well as people’s perception of the quality of primary healthcare services. Method: The national-level data (e.g., the numbers of primary health institutions, personnel, beds, visits, and hospitalizations) regarding primary health institutions were collected from the Health Statistics Yearbook, and the perceptions of the quality of primary healthcare services were collected by a web-based questionnaire survey using an internationally recognized assessment tool (i.e., PCAT-AE). In total, 10,850 persons were surveyed, and 10,419 participants were incorporated into the final analysis after removing invalid questionnaires. A descriptive statistical analysis (i.e., frequency and percentage) was used to analyze the national-level characteristics of primary health institutions and people’s perceptions of the quality of primary healthcare services. Moreover, a logistic regression model was used to analyze the factors influencing the perceptions of the quality of primary healthcare services. Results: From the macro perspective, the number of primary health institutions, beds, and personnel per 10 thousand residents slightly increased from 2013 to 2020, especially in the eastern and central areas. However, the average number of visits and the hospitalization rate in primary health institutions showed a decrease, especially in central and eastern areas. Among participants, 92.2% (9606/10,419) of them had previously sought healthcare services in primary health institutions, and most were seeking general outpatient services (57.06–63.45%), followed by medicine purchasing (16.49–21.51%), physical examinations (9.91–11.49%), preventive health services (5.11–6.48%), and hospitalization services (3.17–5.67%). The total perception scores on the quality of primary healthcare services reported by the participants were 26.19 and 27.00 for rural and urban areas, respectively, which accounted for 65.5% and 67.5% of the total score, respectively, and 26.62, 26.86, and 25.89 for the eastern, central, and western areas, respectively, with percentages of 66.6%, 67.2%, and 64.7%. The perception score on the quality among people contracted with a family doctor (29.83, 74.58%) was much higher than those who were not (25.25, 63.13%), and the difference was statistically significant (*p* < 0.001). Moreover, people who were female, married, had higher incomes, and were diagnosed with various diseases had better perceptions of the primary healthcare services compared to their counterparts (*p* < 0.05). Conclusion: Improvements were seen for primary health institutions, especially in terms of hardware resources such as beds and personnel. However, the service utilization in primary health institutions did not improve between 2013 and 2020. The perception score on the quality of primary healthcare was moderate to low in rural and urban as well as eastern, central, and western areas, but it was significantly higher among people contracted with a family doctor than those who were not. Therefore, it is important for policy makers to take or adjust measures focusing on quality improvement and increasing the service utilization in primary health institutions with good first contact, accessibility, continuity, comprehensiveness, and coordination, such as raising the enrollment rate of family doctors and promoting the provision of high-quality services.

## 1. Introduction

With the increasing prevalence of chronic noncommunicable diseases (e.g., hypertension, diabetes, and cancer) and emerging communicable diseases (e.g., COVID-19), health systems worldwide face significant burdens [1,2]. To protect human health, primary health institutions have been given a high priority, especially in low- and middle-income countries with limited health resources [3]. In China, to provide patients with access to primary health institutions that are close to their residence, a series of favorable policies have been issued. For example, the government increased its subsidies to primary health institutions from CNY 104 billion (USD 15.8 billion) in 2013 to CNY 248 billion (USD 37.6 billion) in 2020 [4,5]. Moreover, the hierarchical diagnosis and treatment system, which encourages patients to visit secondary hospitals after a referral by doctors from primary health institutions, places primary health institutions in the core position to bridge healthcare with other sectors. The key to the success of a hierarchical diagnosis and treatment system also lies in the provision of primary healthcare [6,7]. In addition, access to primary care might be of considerable importance in terms of delivering preventive medical interventions for the major causes of mortality, including cancer and cardiovascular disease. To avoid crowding in higher-level hospitals, the COVID-19 pandemic may also lead to the encouragement of patients seeking healthcare in primary health institutions [1,8].

From the international perspective, it showed that the perception of primary healthcare quality varied with the context and perspective of stakeholders [9,10]. Different stakeholders (i.e., professionals, patients, healthcare managers, and policy makers) use different indicators to evaluate quality [9,11,12]. For example, healthcare professionals pay more attention to the organizational quality orientation, leadership, and satisfaction [13,14]. Patients consider doctor–patient relationships and the adequacy of waiting times to be important [15,16]. In China, some quantitative studies evaluated the quality of primary care among patients aged 18 years and the quality differences by types of healthcare facilities in small-scale areas almost ten years ago [17,18,19]. However, considering the economic development in areas and that the signing of family doctor contracts continues to deepen, people’s perception of the quality of primary healthcare services may vary.

Moreover, it is thus clear that primary healthcare has experienced a long period of development since the Alma Ata Declaration over 40 years ago in 1978 [20]. Therefore, we aim to assess the development of primary health institutions and patients’ perceptions of the quality of primary healthcare services under a variety of measures of promoting primary healthcare. People’s values and expectations matter for the assessment of provider performance and the gaining of the people’s trust, which can provide evidence for improvements in policy and practice to ensure more efficient delivery of high-quality primary healthcare.

## 2. Materials and Methods

### 2.1. Data Collection and Measurements

Considering some cities started to introduce and carry out the hierarchical diagnosis and treatment system in 2014, we collected the macroscopic data of primary health institutions in 2013 and 2020 from the China Health Statistics Yearbook [4,5] to analyze the changes in primary health institutions under the hierarchical diagnosis and treatment system. The data included the number of primary health institutions, the personnel and beds of primary health institutions, the number of visits, and hospitalization. Perceptions of the quality of primary healthcare services were collected by a web-based questionnaire survey.

All people could fill out the questionnaires using electronic equipment (e.g., a mobile phone, laptop, computer, or iPad) based on Credamo, which is a professional online survey platform that works similarly to MTurk in providing research services in China. The inclusion criteria of participants were: (1) people who were more than 18 years old and (2) willing to participate in the study. The exclusion criteria were: (1) people who were under 18 years old and (2) people who did not pass the “attention check” in the questionnaire, which was set to identify careless respondents and improve the data quality. The data were first checked for quality based on the “attention check” in the questionnaire. Then, the logic check and missing values check were carried out, and 431 cases were deleted, which did not bias the final results. Therefore, 10,419 participants were incorporated into the final analysis. The information included in the questionnaire contained: (1) social demographic characteristics, including gender, age, marital status, monthly income, education, occupation, residential city, household registration, and medical insurance; (2) health-related information, which included having been diagnosed with various diseases and a self-perceived health score; and (3) perceptions of the quality of primary healthcare service, which were evaluated by an internationally recognized assessment tool called the Primary Care Assessment Tool-Adult Edition(PCAT-AE) [19,21,22,23,24,25]. The PCAT-AE was designed to assess people’s perception of primary healthcare service quality, and it is an internationally recognized assessment tool that can be compared among countries. The PCAT-AE measured the five core dimensions of primary healthcare services (i.e., first contact, accessibility, continuity, comprehensiveness, and coordination), and it was shown to have good reliability and validity in China [17,26]. Each item has a five-point ordinal response scale with the scoring of 1 = definitely not, 2 = probably not, 3 = probably, and 4 = definitely, and not sure/do not know was assigned a score of 2.5 based on the PCAT-AE manual. Higher scores indicate better perceptions of healthcare services provided by primary health institutions.

### 2.2. Statistical Analysis

A descriptive statistical analysis (i.e., frequency and percentage) was used to analyze the characteristics of primary health institutions, visits to primary health institutions, and perceptions of the quality of primary healthcare services. The number of beds and personnel per 10 thousand residents, the average number of visits, and the hospitalization rate in primary health institutions were calculated based on the number of permanent residents per province/city. We analyzed whether the visits and perceptions of the quality differed significantly among subgroups of the sample using the Mann–Whitney *U* and Kruskal–Wallis tests. Moreover, a logistic regression model was used to analyze the factors influencing the quality perceptions of primary healthcare services. All data analyses were based on the statistical software SPSS 23.0 (IBM, Armonk, NY, USA). Variables with *p* < 0.05 were considered statistically significant.

## 3. Results

**Figure 1:**
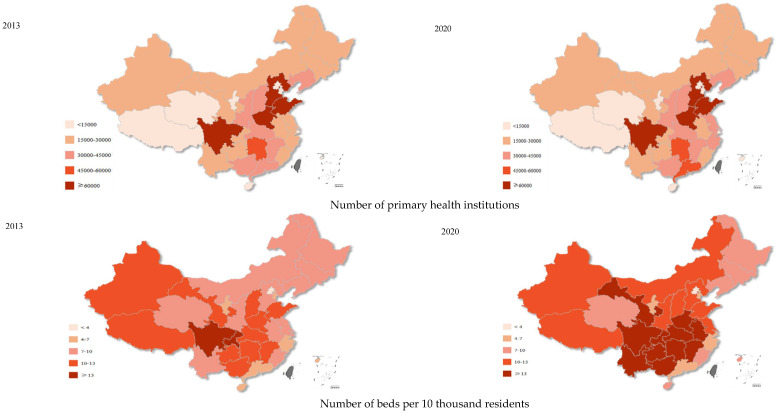

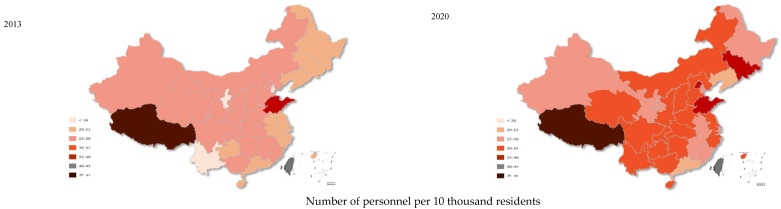
The total number of primary health institutions and the number of beds and personnel per 10 thousand residents in primary health institutions in China between 2013 and 2020.

**Figure 2:**
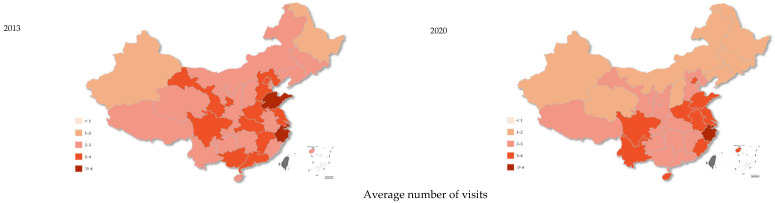

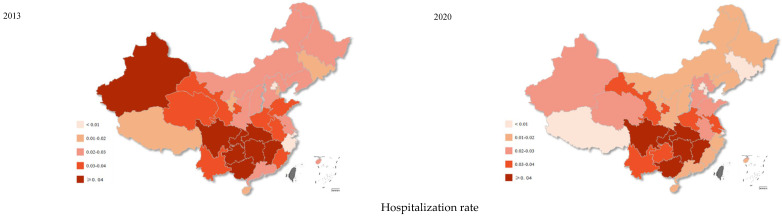
The average number of visits and the hospitalization rate in primary health institutions in China between 2013 and 2020.

From the macro perspective, the number of primary health institutions increased from 2013 to 2020, which raised from 30,000–45,000 to 45,000–60,000 for some eastern areas and from 15,000–30,000 to 30,000–45,000 for some central areas (Figure 1). The number of beds and personnel in primary health institutions per 10 thousand residents had an obvious increase across China, especially in the central areas after the implementation of the hierarchical diagnosis and treatment system. However, the average number of visits and the hospitalization rate in primary health institutions showed a decrease, especially in the central and eastern areas of China (Figure 2).

Table 1 shows the social demographic characteristics of participants by household registration and economic region. Among participants, 92.2% (9606/10,419) of people had sought healthcare services in primary health institutions, with the remaining 7.8% (813/10,419) having never sought such services (Table 2). The proportion of those who had sought healthcare services in primary health institutions was slightly higher in rural areas (94.26%) than that of urban areas (90.4%). From the perspective of economic regions, the proportion of those who had sought healthcare services in primary health institutions in western areas (90.73%) was slightly lower than that of eastern (92.2%) and central areas (92.74%).

**Figure 3:**
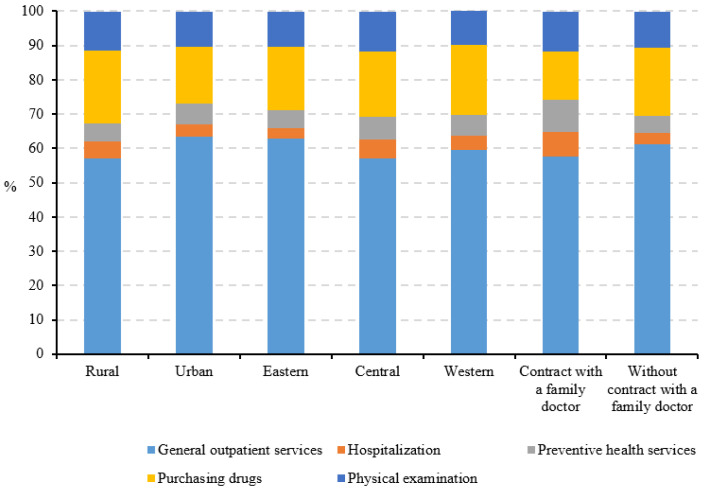
Health services received in primary health institutions.

For the people who had received healthcare services in primary health institutions (Figure 3), most were seeking general outpatient services (57.06–63.45%), followed by medicine purchasing (16.49–21.51%), physical examinations (9.91–11.49%), preventive health services (5.11–6.48%), and hospitalization services (3.17–5.67%). There were no obvious differences in the types of healthcare services received from primary health institutions by household registration and economic region. However, the percentage of preventive and hospitalization health services received by people contracted with a family doctor was much higher (16.67%) than those who were not (8.24%).

**Figure 4:**
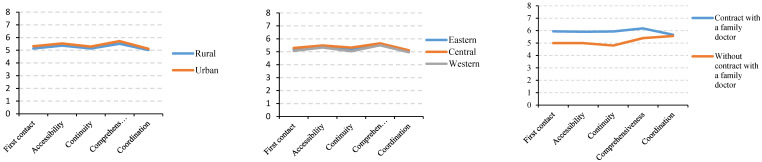
Perception score on the quality of primary healthcare attributes reported by participants.

**Figure 5:**
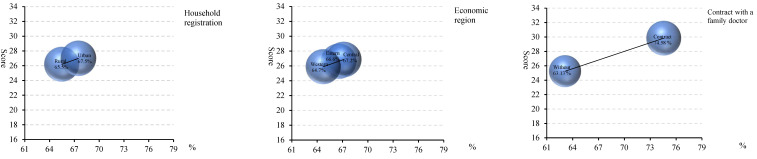
Total perception score on the quality of primary healthcare by household registration, economic region, and whether contracted with a family doctor.

Figure 4 shows the perception score on the quality of primary healthcare attributes. The scores of the five attributes were almost same, and there were no obvious differences by household registration or economic region. However, they were higher among people contracted with a family doctor than who were not. The total perception scores reported by the participants were 26.19 and 27.00 for rural and urban areas, respectively (Figure 5), which accounted for 65.5% and 67.5% of the total score, respectively. By economic region, it was 26.62, 26.86, and 25.89 for the eastern, central and western areas, with the rates of 66.6%, 67.2%, and 64.7%. The perception score on the quality among people contracted with a family doctor (29.83, 74.58%) was much higher than those who were not (25.25, 63.13%).

Table 3 showed that there were significant differences in the perception of the quality of primary healthcare by gender, marital status, income, and whether a family doctor was involved (*p* < 0.05). People who were married (*p* < 0.001, B = 1.575, 95% CI: 1.196, 1.954) and those who contracted with a family doctor (*p* < 0.001, B = 3.309, 95% CI: 2.991, 3.627) had a better perception of primary healthcare services compared to their counterparts. In addition, the perception score on healthcare services quality among people diagnosed with certain diseases was also significantly higher than that of healthy people (*p* < 0.001, B = 0.684, 95% CI: 0.408, 0.960). However, the differences were not significant between urban and rural (*p* = 0.525, B = 0.104, 95% CI: −0.217, 0.425) or the eastern and western areas (*p* = 0.095, B = −0.293, 95% CI: −0.637, 0.051).

## 4. Discussion

In China, improvements were seen for primary health institutions between 2013 and 2020, especially in terms of hardware resources such as beds and personnel. From the perspective of economic regions, the growth in the central areas was obvious, as the number of personnel for almost all central areas increased from 25–30 to 30–35 per 10 thousand residents between 2013 and 2020. This may represent the phenomenon that health resources in primary health institutions were much richer in eastern areas, which was gradually changing with the continuous health investment in the central and western areas in China. Moreover, the investment in personnel for primary healthcare was important, considering that evidence has shown that the increased supply of primary care personnel was associated with improved population health and reduced mortality [27]. However, the average number of visits to primary health institutions decreased from 2013 to 2020, especially in the central and eastern areas. Outpatient services for primary health institutions decreased by 4.8% [4,5]. Although this may be affected by the COVD-19 pandemic, it increased by 21.2% for higher-level hospitals according to the China Health Statistics Yearbook [4,5]. This shows that the primary health institutions have failed to share the growing medical demand, which may be caused by many reasons. First, residents have chosen higher-level hospitals, regardless of serious or minor diseases for a long time in China, and the ability of doctors in primary health institutions is uneven, which may further lead to the distrust in primary healthcare. Second, the expansion of higher-level hospitals with an increasing number of beds may be partly siphoning patients from primary health institutions. Third, technological progress, such as telemedicine, may also indirectly reduce the number of visits to primary health institutions. Moreover, primary healthcare services provided by social capital and the superior hospital were not incorporated in our visit data. Therefore, the decline in visits to primary health institutions does not necessarily mean that the hierarchical diagnosis and treatment system was not effective. However, it may also partly show that people saw doctors in a more sporadic manner rather than following the hierarchical diagnosis and treatment principles, considering the decreasing visits in primary health institutions and the significant increase in higher-level hospitals.

In total, primary health institutions in China accounted for 55.33% of outpatient healthcare (4.11 billion visits) in 2020 [2]. Consistent with this result, our study also showed that more than half of participants received general outpatient care in primary health institutions, which was because primary health institutions mainly provide generalist clinical care (e.g., general outpatient care), testing (e.g., routine blood tests, urine tests, electrocardiography, chest X-rays, and blood glucose tests), and basic public health services (e.g., common chronic disease management and infectious disease prevention) in China [28,29,30]. However, the most economical and effective health strategy—preventive health services—accounted for a low percentage among people receiving primary healthcare services. This may imply that primary health institutions need to further change the service objectives from passive diagnosis and treatment services to the active prevention of health risk factors. At the macro level, the healthy China 2030 plan has put prevention healthcare as a scientific strategy to improve people’s overall heath and continuously improve people’s sense of wellbeing, happiness, and security. Therefore, the system of prevention should be further refined and improved in primary health institutions, at least in terms of the regulation of nutrition, the promotion of fitness, and the regulation of addictive products, especially in relation to aging and behavioral changes.

The perception of the quality of healthcare services is important for the continuous improvement of service delivery and outcomes, especially for primary healthcare, which received a lot of policy prioritization in China. In our study, the reported perception score of quality was mostly medium to low and accounted for just 66.3% of the total score. Although the attribute of comprehensiveness was rated relatively high compared to first contact, accessibility, continuity, and coordination, the scores for all attributes of primary healthcare were not high. In addition, previous studies showed that there were significant differences in the quality of primary healthcare among different areas, mainly due to the economic impact [28,31]. However, we found that the perception scores of quality were all medium to low by household registration and economic region, and the differences were not significant (*p* > 0.05). This may imply that, even though China has increased investment in primary health institutions and hardware conditions have improved since 2013, its quality of primary healthcare still needs to be substantially strengthened.

However, we found that the perception of the quality of primary healthcare services were much better in patients who were contracted with a family doctor than in those who were not (*p* < 0.001). This is consistent with previous studies that reported that those who reported a higher quality of primary care received care from a family doctor or general practice [19,32]. This implies that primary healthcare will be strengthened when services are organized better by having a family doctor. Therefore, a family doctor assignment system should be further promoted to ensure a trusting relationship between primary medical teams and residents and to provision high-quality primary healthcare services.

In our study, age did not influence the level of perception of the quality of primary healthcare (*p* > 0.05). This result was different from previous research, which reported that a greater age was positively correlated a positive perception of quality [33]. We also looked at the association between the perception of the quality of care and health status. People who had been diagnosed with certain diseases, who were more in need of healthcare services, typically had a better perception of the quality of primary healthcare services. In addition, compared with those only completing junior high school or below, participants with a higher education degree (i.e., postgraduate or above) showed a lower perception of quality regarding primary health services (*p* = 0.024). This finding is consistent with a previous study that found that a lower level of education is associated with greater satisfaction in health services [33,34]. This may be because people with a higher education degree apply higher standards in assessing the healthcare services received so that even objectively better healthcare services do not meet their subjective standards of care [33].

## 5. Conclusions

Improvements were seen for primary health institutions, especially in terms of hardware resources such as numbers of beds and personnel. However, the service utilization in primary health institutions did not improve between 2013 and 2020. The perception score for the quality of primary healthcare was moderate to low in rural and urban as well as eastern, central, and western areas, but it was significantly higher among people who were contracted with a family doctor than those who were not. Therefore, it is important for policy makers to take or adjust measures focusing on quality improvement and increasing the service utilization in primary health institutions with good first contact, accessibility, continuity, comprehensiveness, and coordination, such as raising the enrollment rate of family doctors and promoting the provision of high-quality services.

## 6. Limitations

This study is subject to some limitations. First, considering the differences in economics, traditional cultures, and health service development among areas, this study may not represent the whole of China, although we collected data from more than ten thousand people. Second, although we removed the data that did not meet our quality standards from the online questionnaire, the results may also be subject to the limitations of online investigations. Third, considering the influences of technological progress and patients’ long-term medical behavior, the decline in visits to primary health institutions does not necessarily mean the hierarchical diagnosis and treatment system or the national policy of strengthening primary healthcare is not effective. Fourth, the macro-level data regarding primary health institutions did not include the primary healthcare services provided by social capital and the superior hospital. Fifth, although the COVID-19 epidemic was under control in China when the investigation was conducted, the epidemic was still sporadic, which may partly influence our results. In addition, some confounders (e.g., education and income) may influence the results. Despite these limitations, the findings from this study are helpful in informing policy decisions on primary healthcare and practice, especially in the context of aging, the building of a healthy China in 2030, and the hierarchical diagnosis and treatment system in China.

## Figures and Tables

**Table 1:** Social demographic characteristics of participants.

	Household Registration				Economic Region					
Items	Urban		Rural		Eastern		Central		Western	
	N	%	N	%	N	%	N	%	N	%
Gender										
Male	2343	42.14	2375	48.88	2082	43.43	1705	49.49	797	43.7
Female	3217	57.86	2484	51.12	2712	56.57	1740	50.51	1027	56.3
Marital status										
Married	3310	59.53	1776	36.55	2405	50.17	1672	48.53	782	42.87
Unmarried	2199	39.55	3042	62.61	2351	49.04	1740	50.51	1026	56.25
Divorced	46	0.83	34	0.7	33	0.69	31	0.9	11	0.6
Widowed	5	0.09	7	0.14	5	0.1	2	0.06	5	0.27
Age										
<60	5508	99.06	4831	99.42	4763	99.35	3416	99.16	1808	99.12
>60	52	0.94	28	0.58	31	0.65	29	0.84	16	0.88
Monthly income (CNY)										
<3000	982	17.66	1639	33.73	1037	21.63	985	28.59	533	29.22
3000~5000	1265	22.75	1374	28.28	1175	24.51	890	25.83	502	27.52
5000~10,000	2312	41.58	1534	31.57	1921	40.07	1176	34.14	609	33.39
10,000~30,000	913	16.42	289	5.95	600	12.52	367	10.65	161	8.83
30,000~50,000	72	1.29	15	0.31	48	1	21	0.61	15	0.82
≥50,000	16	0.29	8	0.16	13	0.27	6	0.17	4	0.22
Education										
Junior high school or below	65	1.17	162	3.33	102	2.13	76	2.21	40	2.19
Senior high school	542	9.75	837	17.23	655	13.66	457	13.27	229	12.55
Undergraduate or college graduate	4235	76.17	3536	72.77	3527	73.57	2605	75.62	1377	75.49
Postgraduate or above	718	12.91	324	6.67	510	10.64	307	8.91	178	9.76

**Table 2:** Health services seeking in PHIs among participants.

Items			Ever Sought Services in PHIs	Never Sought Services in PHIs
Total		Frequency	9606	813
		%	92.20	7.80
Household registration	Rural	Frequency	4580	279
		%	94.26	5.74
	Urban	Frequency	5026	534
		%	90.40	9.60
	*p* value		<0.001	
Economic region	Eastern	Frequency	4420	374
		%	92.20	7.80
	Central	Frequency	3195	250
		%	92.74	7.26
	Western	Frequency	1655	169
		%	90.73	9.27
	*p* value		0.035	

PHIs: Primary health institutions.

**Table 3:** Factors influencing the perception of the quality of primary healthcare services.

Variables	B	SE	*p*	95% CI
Gender				
Male	0.314	0.13	0.015	0.060~0.568
Female (reference)				
Age				
<16 (reference)				
16–29	0.256	1.298	0.843	−2.287~2.799
30–39	0.169	1.306	0.897	−2.391~2.729
40–49	0.177	1.319	0.893	−2.407~2.762
50–59	0.225	1.341	0.867	−2.402~2.853
≥60	0.899	1.464	0.539	−1.971~3.769
Monthly income				
<3000 (reference)				
3000–5000	1.31	0.196	<0.001	0.927~1.693
5000–10,000	2.404	0.203	<0.001	2.006~2.802
10,000–30,000	3.467	0.27	<0.001	2.936~3.997
30,000–50,000	2.909	0.716	<0.001	1.507~4.312
>50,000	−0.195	1.361	0.886	−2.863~2.473
Education				
Junior high school or below (reference)				
Senior high school	−0.012	0.462	0.979	−0.918~0.893
Undergraduate or college graduate	−0.078	0.447	0.862	−0.954~0.799
Postgraduate or above	−1.111	0.493	0.024	−2.077~−0.145
Marital status				
Unmarried (reference)				
Married	1.575	0.193	<0.001	1.196~1.954
Divorced	−1.329	0.808	0.100	−2.912~0.254
Widowed	0.634	1.842	0.731	−2.976~4.244
Household registration				
Rural (reference)				
Urban	0.104	0.164	0.525	−0.217~0.425
Medical insurance				
NRCMI *	1.36	0.189	<0.001	0.989~1.731
BMIUR *	0.887	0.165	<0.001	0.564~1.210
BMIRE *	0.436	0.179	0.015	0.086~0.787
Commercial health insurance	0.356	0.196	0.069	−0.028~0.741
No health insurance (reference)				
Self-reported health score	0.63	0.049	<0.001	0.535~0.725
Prevalence				
Yes	0.684	0.141	<0.001	0.408~0.960
No (reference)				
Distance to medical institutions				
<1 km (reference)				
1–2 km	0.442	0.572	0.439	−0.679~1.564
2–3 km	0.618	0.47	0.189	−0.304~1.539
3–4 km	0.473	0.436	0.278	−0.382~1.327
4–5 km	0.513	0.421	0.223	−0.312~1.338
>5 km	0.492	0.414	0.235	−0.320~1.303
Contracted with a family doctor				
Yes	3.309	0.162	<0.001	2.991~3.627
No (reference)				
Economic region				
Eastern (reference)				
Central	0.36	0.141	0.011	0.084~0.636
Western	−0.293	0.176	0.095	−0.637~0.051

* NRCMI: New rural cooperative medical insurance; BMIUR: Basic medical insurance for urban residents; BMIRE: Basic medical insurance for urban employees; CMI: Commercial medical insurance.

## Data Availability

All of the main data were included in the results. Additional materials with details may be obtained from the corresponding author.
